# *Vogesella perlucida*-induced bacteremia in an advanced-age patient: first case report

**DOI:** 10.1186/s12879-020-05420-w

**Published:** 2020-09-18

**Authors:** Zengxian Yu, Fang Zhu, Xinghe Tao, Lu Zhang, Suliu Wu, Chunfu Dong, Yeqing Dong, Ge chen, Xinyang Zhou, Yinfei Fang, Kechen Xu

**Affiliations:** 1 Clinical Laboratory Center, Wuyi First People’s Hospital, Wuyi, Jinhua, 321200 Zhejiang China; 2 Wuyi First People’s Hospital, Wuyi, Jinhua, 321200 Zhejiang China; 3 Pathology department, Jinhua Central Hospital, Jinhua, 321000 Zhejiang China

**Keywords:** *Vogesella perlucida*, Bacteremia, 16S rRNA gene sequence analysis

## Abstract

**Background:**

*Vogesella* species are common aquatic, Gram-negative rod-shaped bacteria, originally described in 1997. *Vogesella perlucida* was first isolated from spring water in 2008. Furthermore, bacterial pathogenicity of *Vogesella perlucida* has never been reported. Here, we report the first case of rare *Vogesella perlucida*-induced bacteremia in an advanced-age patient with many basic diseases and history of dexamethasone abuse.

**Case presentation:**

A 71-year-old female was admitted with inflamed upper and lower limbs, rubefaction, pain and fever (about 40 °C). She had been injured in a fall at a vegetable market and then touched river snails with her injury hands. A few days later, soft tissue infection of the patient developed and worsened. Non-pigmented colonies were isolated from blood cultures of the patient. Initially, *Vogesella perlucida* was wrongly identified as *Sphingomonas paucimobilis* by Vitek-2 system with GN card. Besides, we failed to obtain an acceptable identification by the MALDI-TOF analysis. Finally, the isolated strain was identified as *Vogesella perlucida* by 16S rRNA gene sequences. In addition, the patient recovered well after a continuous treatment of levofloxacin for 12 days.

**Conclusion:**

Traditional microbiological testing system may be inadequate in the diagnosis of rare pathogenic bacteria. Applications of molecular diagnostics techniques have great advantages in clinical microbiology laboratory. By using 16S rRNA gene sequence analysis, we report the the first case of rare *Vogesella perlucida*-induced bacteremia.

## Background

*Vogesella* species belong to the family Neisseriaceae, order Neisseriales, class Betaproteobacteria, mainly characterized as Gram-negative, rod- shaped, aerobic or facultatively anaerobic and chemoheterotrophic bacteria [1–10]. So far as we know, there are no reported cases of human disease caused by *Vogesella* species. Although *Vogesella urethralis* has been isolated from human urine, its pathogenicity has not been reported [10]. *Vogesella perlucida* was first described in 2008 when it was discovered from water samples collected from a spring located in Tainan County, Taiwan [2]. *Vogesella perlucida* is aerobic, non-pigmented, Gram-negative, non-spore-forming and rod-shaped [2]. *Vogesella perlucida* is positive for cytochrome oxidase, catalase, lipase (corn oil) and hydrolysis of Tweens 20, 40, 60, 80 and negative for DNase and for hydrolysis of starch, chitin and casein [2]. Reviewing the previous literature, we found no relevant reports of *Vogesella perlucida*-induced infectious diseases.

## Case presentation

On September 13, 2019, a 71-year old female presented at the department of emergency medicine with swollen upper and lower extremities, rubefaction, pain and fever (about 40 °C). Approximately 7 days before outpatient service, the patient had been injured in a fall at a vegetable market and later touched river snails with her injury hands. Three days after the injury, she began to show symptoms and her extremities began to swell and ache. Six days after the injury, she developed a fever but her skin does not fester.

In addition, the patient was admitted to the department of cardiology in July 2019, diagnosed with coronary atherosclerotic heart disease, cardiac function level III, hypertension level III, syncope-first considering vasovagal syncope, chronic bronchitis, chronic infectious arthritis, kidney stone, intracranial atherosclerosis, carotid plaque, lower limb atherosclerosis, gastritis, hyperlipemia and abnormal renal function. This patient has been suffering from chronic bronchitis for more than 10 years, lacking regular diagnosis and treatment. Besides, she has been suffering from rheumatoid arthritis for more than 30 years, with limb joint pain and irregularly taking dexamethasone and painkillers for more than 10 years. According to the pain symptoms, she took painkillers intermittently. Furthermore, she took dexamethasone daily at a dose of 0.75 to 3 mg. The cardiologist gave her effective treatment for her cardiovascular disease and also advised her not to abuse dexamethasone and painkillers. She began taking aspirin enteric-coated tablets, irbesartan tablets, and succinic metoprolol sustained-release tablets on a continuous daily basis. However, she still continued to abuse dexamethasone and painkillers. She complained that she felt weak without dexamethasone.

In this outpatient service, blood examination revealed white blood count (8.9 × 10^9^/L), Neutrophils percentage (83.4%), the absolute neutrophil count (7.4 × 10^9^/L), CRP (10.2 mg/L), PCT (1.53 ng/L). On physical examination, the patient had a temperature of 38.1 °C, heart rate–74 bpm, blood pressure–144/62 mmHg, oxygen saturation–99%. Skull CT showed no significant abnormalities but the CT scan of the lung shows small nodules in the left lung.

**Fig. 1:**
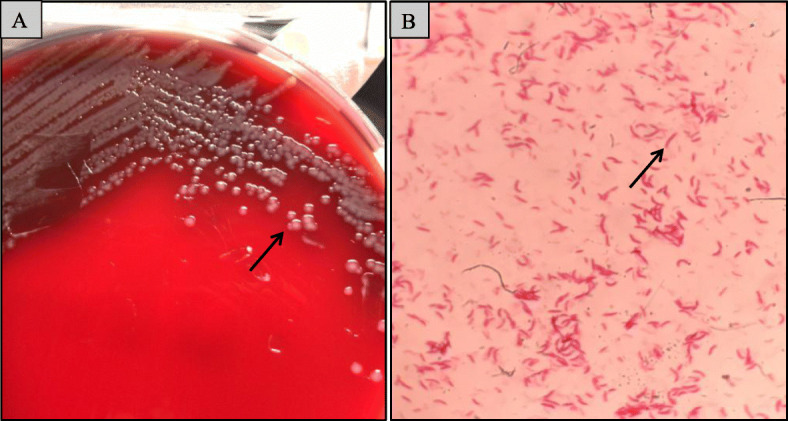
**a**. Isolated strain on the blood agar. The formed colonies are visible, round, entire, convex and colourless to white. **b**. Gram-negative, aerobic, non-spore-forming and rod-shaped bacterium. Total magnification, × 1000

Furthermore, the emergency physician considered deterioration of soft tissue infection and recommended hospitalization. Then, the patient was admitted to hospital and took a further thorough laboratory examination (Table 1). Blood examination revealed white blood count (14.9 × 10^9^/L), Neutrophils percentage (90.6%), the absolute neutrophil count (13.5 × 10^9^/L), hsCRP (163.97 mg/L). The disease progressed rapidly. Two blood cultures were sent for examination. Our laboratory received the first blood culture sample at 16 h40 (Beijing time) on September 13, 2019 and it was collected in the emergency room. The second blood culture sample was collected in the surgical ward and our laboratory received the sample at 21 h52 (Beijing time) on September 13, 2019. As a result, the two blood cultures were both positive for *Sphingomonas paucimobilis* identified by Vitek-2 system with GN card (BioMérieux Inc., France) (Table 2) and the strain was susceptible to most antibiotics (Table 3). Non-pigmented colonies were formed on blood agar subcultured from the blood culture bottle after 20 h of incubation in 5% CO_2_ at 37 °C. But our lab technicians found that the colony morphology (Fig. 1) did not match the *Sphingomonas paucimobilis*, which should be a yellow-pigmented bacillus [11]. So we did MALDI-TOF analysis by mass spectrometer (Brucker Inc.,Germany), but we failed to obtain an acceptable identification.

**Table 1:** Patients selected laboratory results on days of admission and discharge from hospital

Laboratory test	Outpatient tests	Admission tests	Discharge tests	Reference range
WBC[10^9/L]	8.9	14.9	7.8	3.5–9.5
NEUT%[%]	83.4	90.6	74.3	40.0–75.0
ANC[10^9/L]	7.4	13.5	5.8	1.8–6.3
LYMPH%[%]	13.1	6.1	20.4	20.0–50.0
LYMPH[10^9/L]	1.2	0.9	1.6	1.1–3.2
Procalcitonin [ng/ml]	1.53		0.06	0.00–0.50
HsCRP [mg/L]		163.97	23.71	0.00–10.00
CRP [mg/L]	10.2			0.00–10.00
APTT [s]	23.1			25.0–35.0
INR	1.01			0.80–1.50
Bilirubin, total [umol/L]	16.0	18.9		0.0–15.0
Bilirubin conjugated [umol/L]	6.8	9.1		0.0–6.0
Urea [mmol/L]	9.70	9.05		1.70–8.30
LDH [U/L]		404		135–225
CK-MB [U/L]		50		0–24
SAA [mg/L]		286.8		0.0–10.0
D-dimer	991			80–500
NT-pro BNP [ng/L]	1160			300–900
BNP [pg/ml]		435		0–100

**Table 2:** Biochemical profile determined for strain by Vitek 2 GN system

Biochemical reaction	Result	Biochemical reaction	Result	Biochemical reaction	Result
APPA	**–**	BXYL	**–**	SUCT	**+**
ADO	**–**	BAlap	**–**	NAGA	**–**
PyrA	**–**	ProA	**+**	AGAL	**–**
lARL	**–**	LIP	**–**	PHOS	**–**
dCEL	**–**	PLE	**–**	GlyA	**+**
BGAL	**–**	TyrA	**+**	ODC	**–**
H2S	**–**	URE	**–**	LDC	**–**
BNAG	**+**	dSOR	**–**	ODEC	**–**
AGLTp	**+**	SAC	**+**	lHISa	**–**
dGLU	**+**	dTAG	**–**	CMT	**–**
GGT	**–**	dTRE	**+**	BGUR	**–**
OFF	**–**	CIT	**–**	0129R	**–**
BGLU	**–**	MNT	**–**	GGAA	**–**
dMAL	**+**	5KG	**–**	lMLTa	**–**
dMAN	**–**	lLATk	**–**	ELLM	**+**
dMNE	**–**	AGLU	**–**	lLATa	**–**
Identification (% ^*b*^)	Sphingomonas paucimobilis(95)				

^*a*^ **+**, positive; −, negative

^*b*^ Percent confidence of identification

**Table 3:** 16S rRNA gene sequence analysis results

Results	GenBank Accession No.	Sequence Similarity
Vogesella perlucida	NR_044326.1	99.11%
Vogesella mureinivorans	NR_104556.1	98.46%
Vogesella amnigena	NR_137,334.1	97.88%
Sequence:		
TGCAAGTCGAACGGTAACAGGGTGCTTGCACCGCTGACGAGTGGCGAACGGGTGAGTAATGCGTCGGAACGTGCCGAGTAGTGGGGGATAACGCAGCGAAAGTTGTGCTAATACCGCATACGTACTGAGGTAGAAAGTGGGGGACCTTCGGGCCTCACGCTATTCGAGCGGCCGACGTCTGATTAGCTAGTAGGTGAGGTAAAGGCTCACCTAGGCGACGATCAGTAGCGGGTCTGAGAGGATGATCCGCCACACTGGGACTGAGACACGGCCCAGACTCCTACGGGAGGCAGCAGTGGGGAATTTTGGACAATGGGCGAAAGCCTGATCCAGCCATGCCGCGTGTCTGAAGAAGGCCTTCGGGTTGTAAAGGACTTTTGTCAGGGAGGAAATCCCTAGCGTTAATACCGCTGGGGGATGACAGTACCTGAAGAATAAGCACCGGCTAACTACGTGCCAGCAGCCGCGGTAATACGTAGGGTGCGAGCGTTAATCGGAATTACTGGGCGTAAAGCGTGCGCAGGCGGTTTGATAAGCCAGATGTGAAATCCCCGAGCTCAACTTGGGAACTGCGTTTGGAACTGTCAGACTAGAGTGCGTCAGAGGGGGGTGGAATTCCGCGTGTAGCAGTGAAATGCGTAGAGATGCGGAGGAACACCGATGGCGAAGGCAGCCCCCTGGGATGACACTGACGCTCATGCACGAAAGCGTGGGGAGCAAACAGGATTAGATACCCTGGTAGTCCACGCCCTAAACGATGTCAATTAGCTGTTGGGGGTTAGAATCCCTGGTAGCGTAGCTAACGCGTGAAATTGACCGCCTGGGGAGTACGGCCGCAAGGTTAAAACTCAAAGGAATTGACGGGGGCCCGCACAAGCGGTGGATGATGTGGATTAATTCGATGCAACGC

Then we used 16S rRNA gene sequence analysis to identify the isolated strain, which indicated that the isolated strain (GenBank Accession Number MT912571) was closely related to *Vogesella perlucida* (99.11%, GenBank Accession No. NR_044326.1), *Vogesella mureinivorans* (98.46%, GenBank Accession No. NR_104556.1) and *Vogesella amnigena* (97.88%, GenBank Accession No. NR 137334.1) (Table 3). Meanwhile, we compared the traits of this strain with previous literature reports on *Vogesella perlucida* [2] and the characteristic of non-pigment is the biggest difference between *Vogesella perlucida* with other species of *Vogesella* [2]. Finally, we determined that *Vogesella perlucida* was the cause of this bacteremia.

Initially, the patient was treated with a combination of Vancomycin and Levofloxacin. Gradually, the patient’s temperature returned to normal. After the release of microbial diagnosis report and antibacterial spectrum (Table 4) in our laboratory, the treatment regimen was adjusted to apply Levofloxacin only. After 12 days of treatment, the patient recovered well.

**Table 4:** Antibiogram results

Antibiotic	Antibiogram results	MIC^a^
Colistin	Resistant	8
Amikacin	Sensitive	≤2
Aztreonam	Sensitive	≤1
Ceftazidime	Sensitive	≤0.12
Ciprofloxacin	Sensitive	≤0.25
Cefepime	Sensitive	≤0.12
Imipenem	Sensitive	≤0.25
Levofloxacin	Sensitive	≤0.12
Minocycline	Sensitive	≤1
Tobramycin	Sensitive	≤1
Piperacillin/Tazobactam	Sensitive	≤4
Meropenem	Sensitive	≤0.25
Ticarcillin/Clavulanic Acid	Sensitive	≤8
Trimethoprim/Sulfamethoxazole	Sensitive	≤20
Tigecycline	Sensitive	≤0.5
Cefoperazone/Sulbactam	Sensitive	≤8
Doxycycline	Sensitive	≤0.5

^a^*MIC* Minimal inhibitory concentration [μg/L]

## Discussion and conclusion

From the patient’s medical history, the 71-year old patient has many basic diseases, which means that immunity of the patient is relatively poor. In addition, the patient has abused dexamethasone for more than 10 years and long-term treatment with steroids can cause side effects, such as adrenal insufficiency, increased infection risk, hyperglycaemia, high blood pressure, osteoporosis, and so on [12]. As a result, the long-term abuse of dexamethasone makes the patient susceptible to infection. *Vogesella perlucida*, can be easily isolated from fresh water [13, 14]. The progress of touching river snails with her injury hands gives *Vogesella perlucida* a chance to invade the body and cause bacteremia.

From this case, we can suspect that the strain may have some genetic mutation, which would make it more virulent and invasive to infect humans. Latest, reseachers found a novel specie of the genus *Vogesella* with the name *Vogesella urethralis*, isolated from human urine [10]. Interestingly, *Vogesella urethralis* was also wrongly identified as *Sphingomonas paucimobilis* by Vitek-2 system with GN identification card and failed to obtain an acceptable identification by the Vitek MS system [10]. Then the researchers also used 16S rRNA gene sequence analysis to identify the strain and finally revealed that the isolate was *Vogesella urethralis*, with the highest similarities to *V. perlucida* DS-28 T (98.8%) and *V. mureinivorans* 389 T (98.1%).

The identification results of Vitek-2 system will interfere with the accurate identification of *Vogesella* strain. Especially in the case that MALDI-TOF analysis can’t provide an acceptable identification, our diagnostic process is in a dilemma. Therefore, we have to use more advanced technology to identify strains. In conclusion, traditional microbiological testing system, such as Vitek-2 system, may be inadequate in the diagnosis of rare pathogenic bacteria. Applications of molecular diagnostics techniques have great advantages in clinical microbiology laboratory, such as 16S rRNA gene sequence analysis. Finally, the strain isolated from the blood culture of the patient was identified as *Vogesella perlucida* and we report the the first case of rare *Vogesella perlucida*-induced bacteremia.

Moreover, along with intensive and extensive contact with natural environment, human are at a higher risk exposing to more opportunistic pathogen. Human infectious diseases caused by rare opportunistic pathogen are also becoming more common. In the future, it would be a huge challenge to maintain regional and global public health security.

## Data Availability

The datasets used and/or analysed during the current study available from the corresponding author on reasonable request.

## Abbreviations

CRP: C-Reactive protein
CT: Computed tomography
RNA: Ribose Nucleic Acid
MALDI-TOF: Matrix-Assisted Laser Desorption/ Ionization Time of Flight
GN: Gram-Negative
