# Correction to: Vogesella perlucida-induced bacteremia in an advanced-age patient: first case report

**DOI:** 10.1186/s12879-020-05442-4

**Published:** 2020-09-29

**Authors:** Zengxian Yu, Fang Zhu, Xinghe Tao, Lu Zhang, Suliu Wu, Chunfu Dong, Yeqing Dong, Ge Chen, Xinyang Zhou, Yinfei Fang, Kechen Xu

**Affiliations:** 1 Clinical Laboratory Center, Wuyi First People’s Hospital, Wuyi, Jinhua, Zhejiang, 321200 China; 2 Wuyi First People’s Hospital, Wuyi, Jinhua, Zhejiang, 321200 China; 3 Pathology department, Jinhua Central Hospital, Jinhua, Zhejiang, 321000 China

**Correction to: BMC Infect Dis 20, 687 (2020)**

**https://doi.org/10.1186/s12879-020-05420-w**

Following publication of the original article [1], the authors identified errors in some authors’ affiliations.

Below the correct affiliations are given:

- Fang Zhu’s affiliation should be Pathology department, Jinhua Central Hospital, Jinhua 321000, Zhejiang, China;

- Suliu Wu’s affiliation should be Wuyi First People’s Hospital, Wuyi, Jinhua 321200, Zhejiang, China;

-Ge Chen’s affiliation should be Wuyi First People’s Hospital, Wuyi, Jinhua 321200, Zhejiang, China;

- Xinyang Zhou’s affiliation should be Wuyi First People’s Hospital, Wuyi, Jinhua 321200, Zhejiang, China;

- Yinfei Fang’s affiliation should be Pathology department, Jinhua Central Hospital, Jinhua 321000, Zhejiang, China.

The author group has been updated above.
